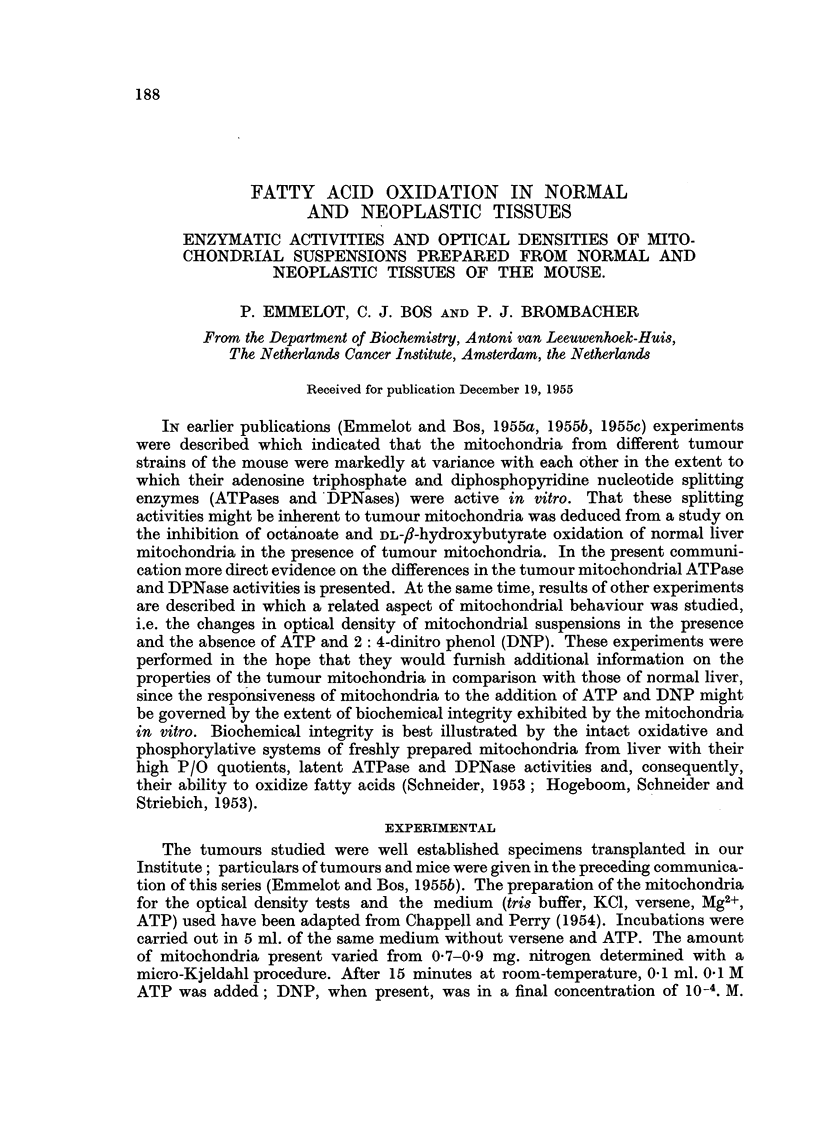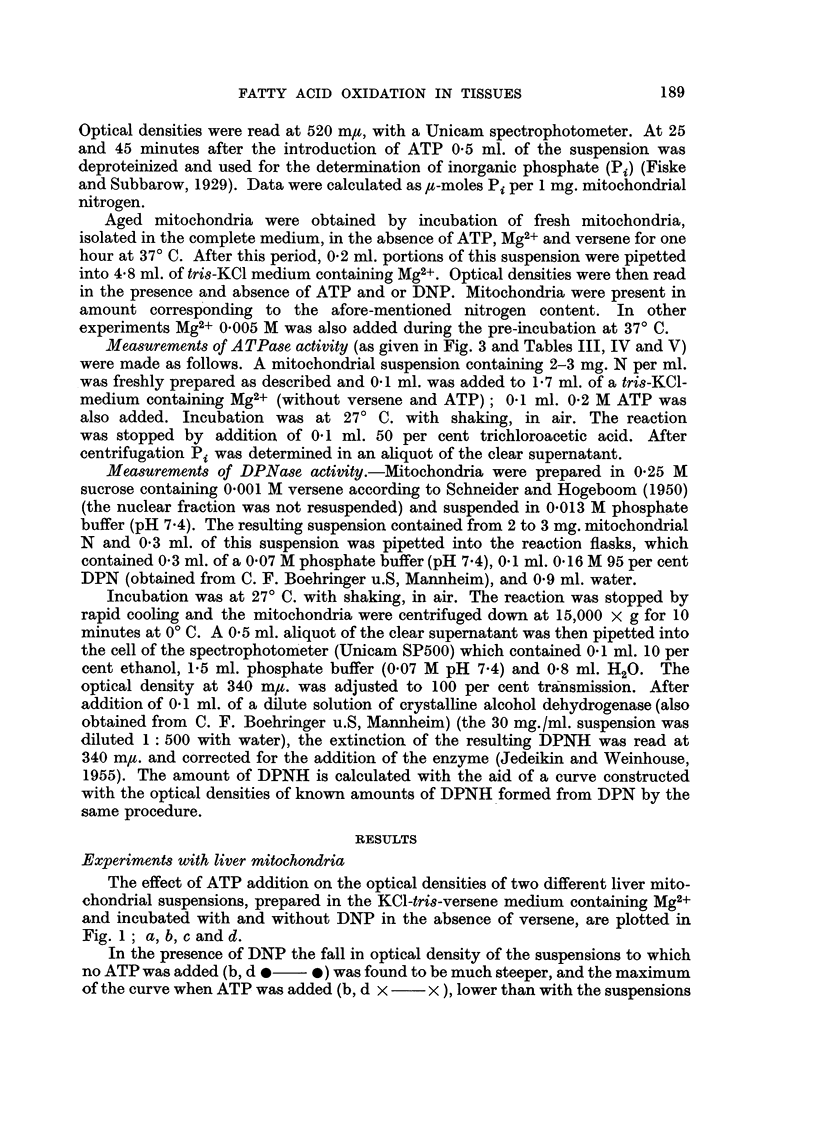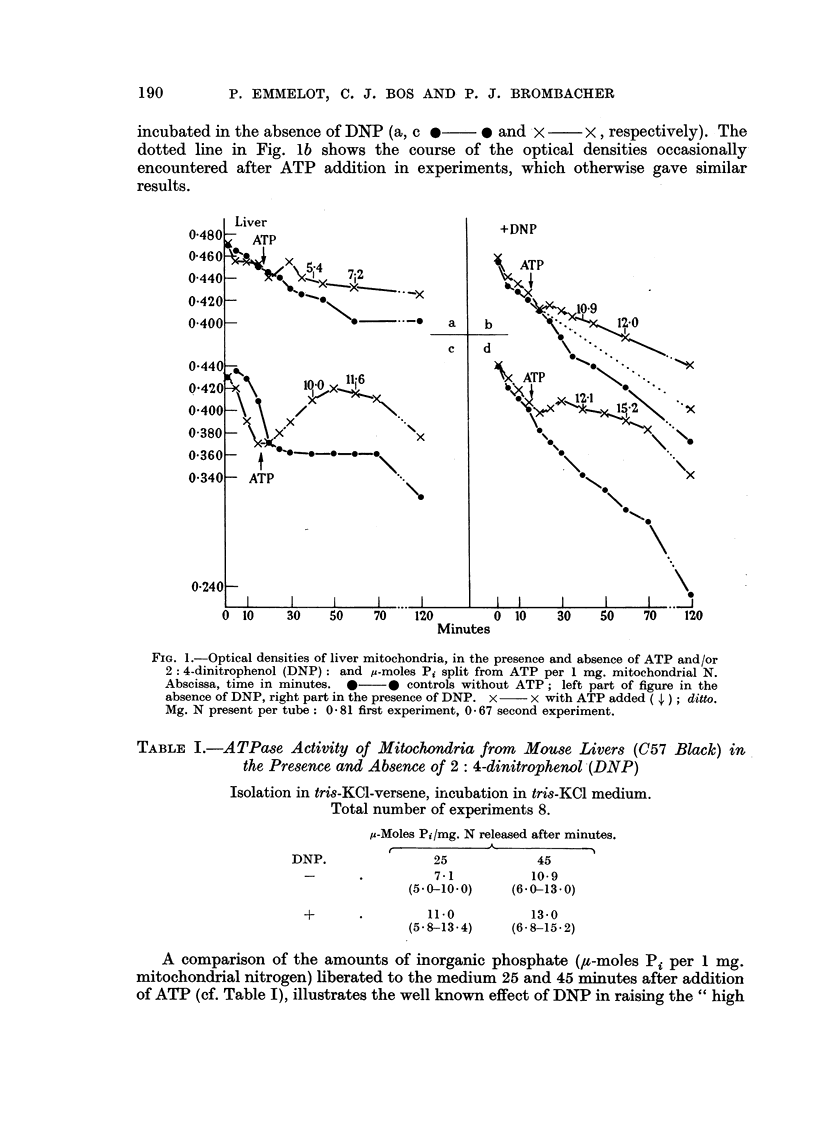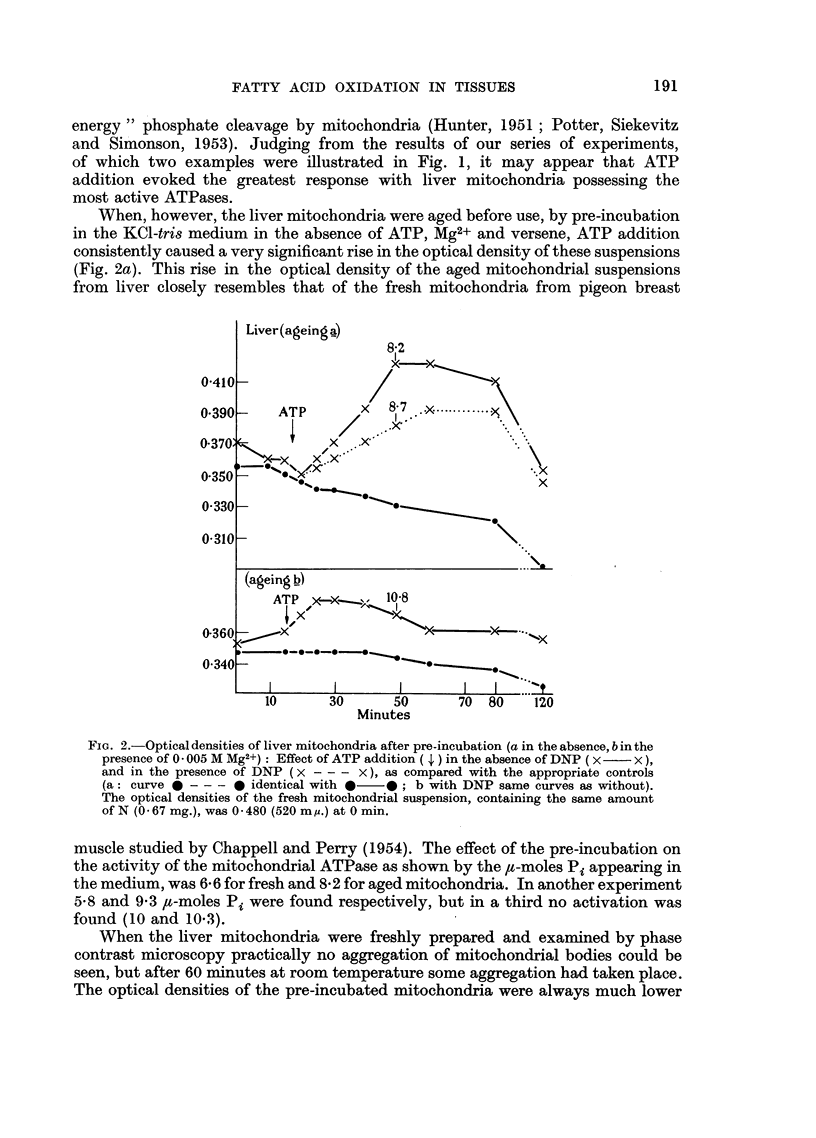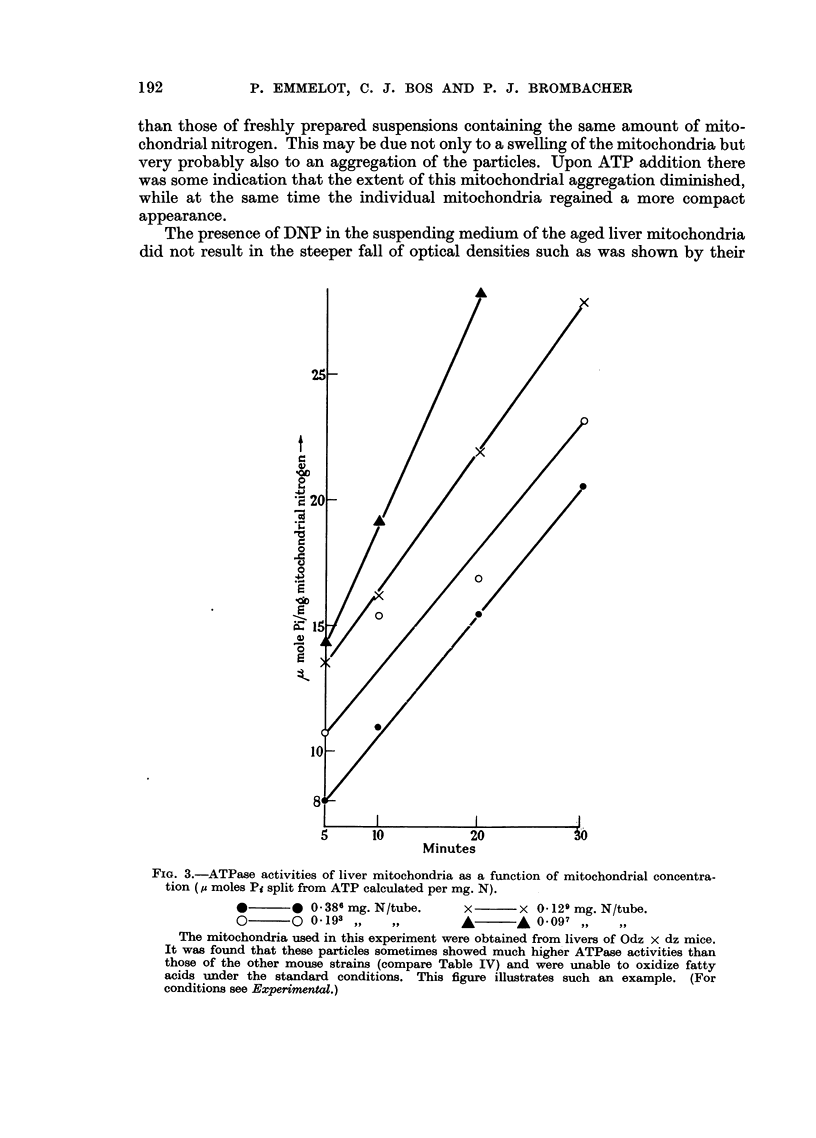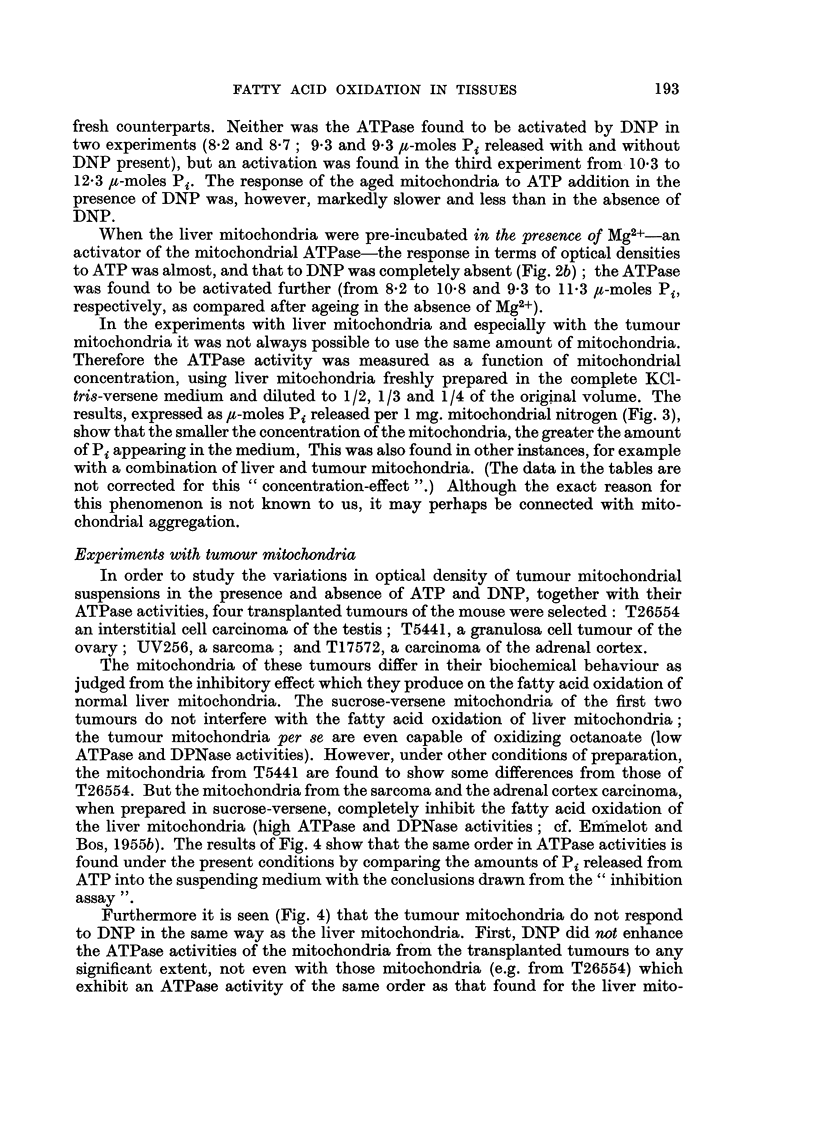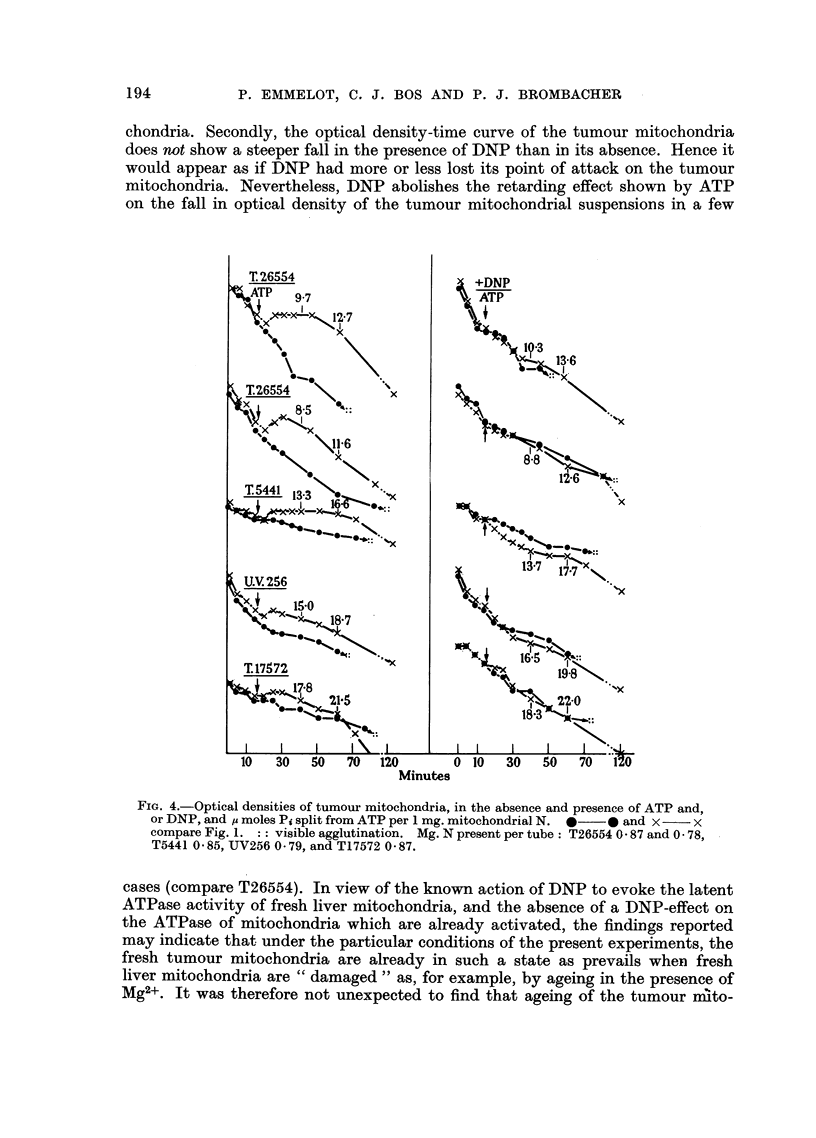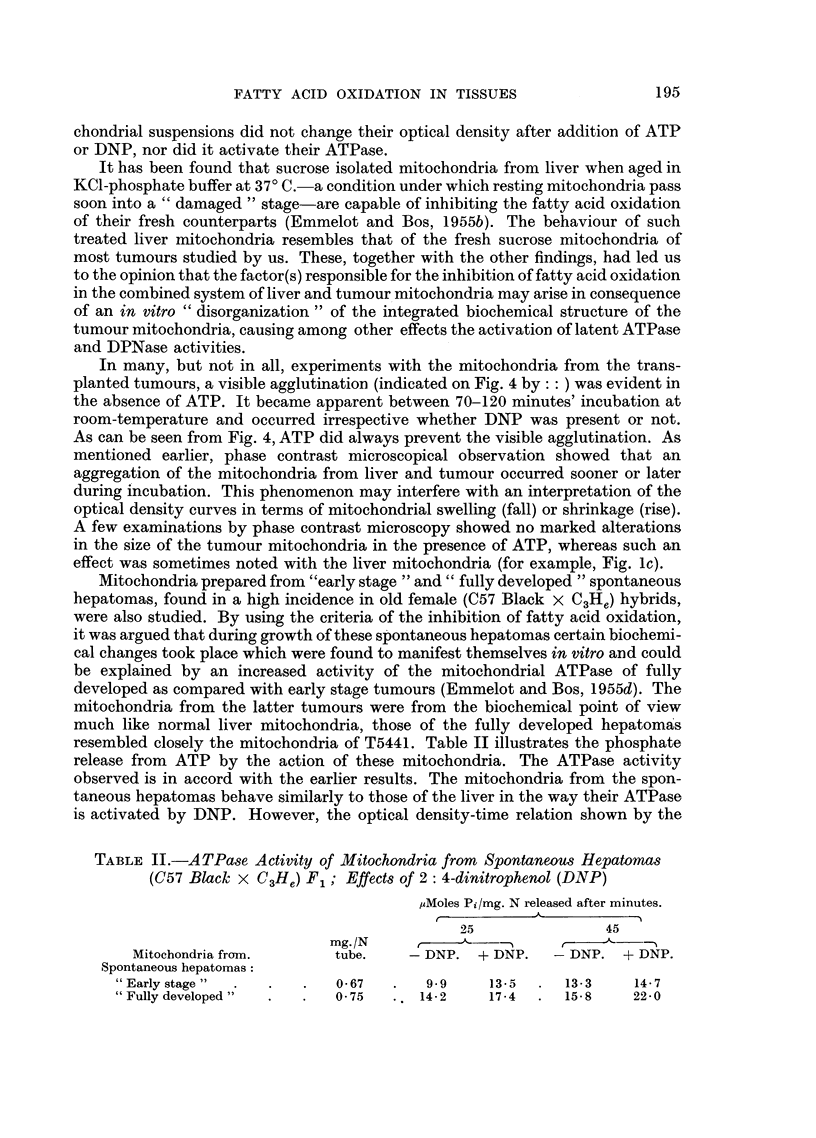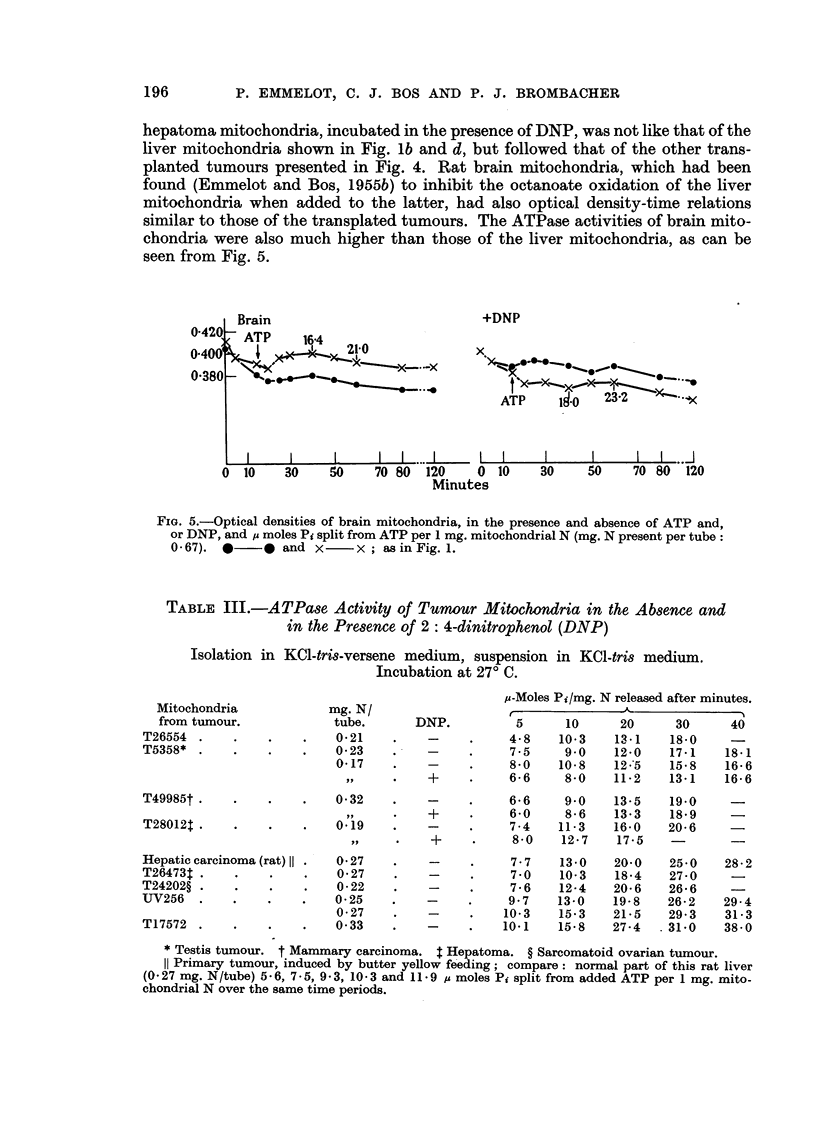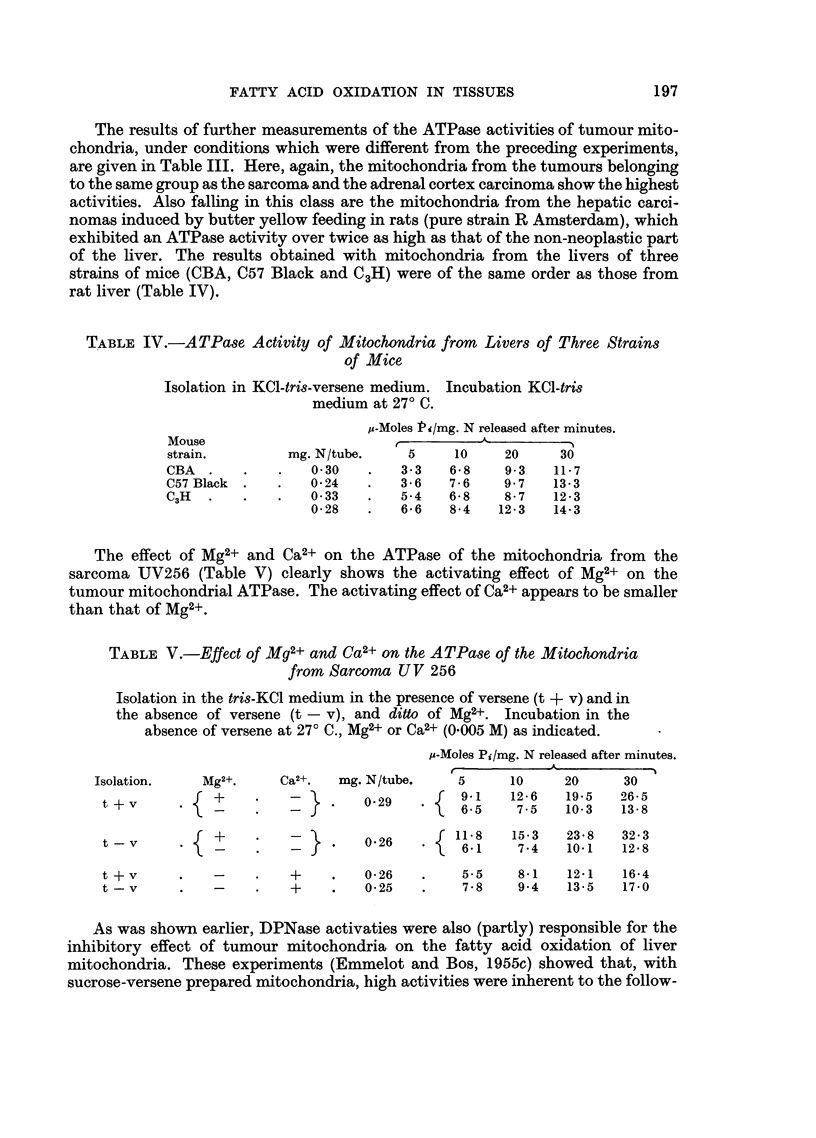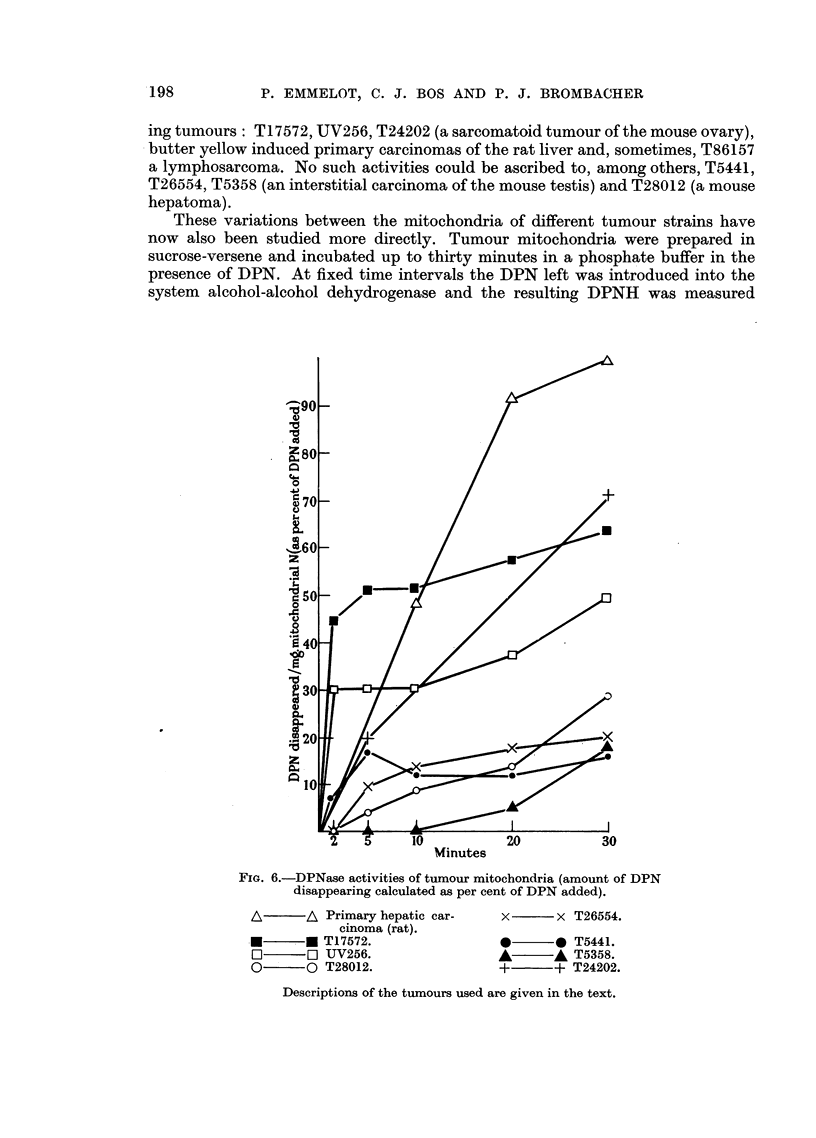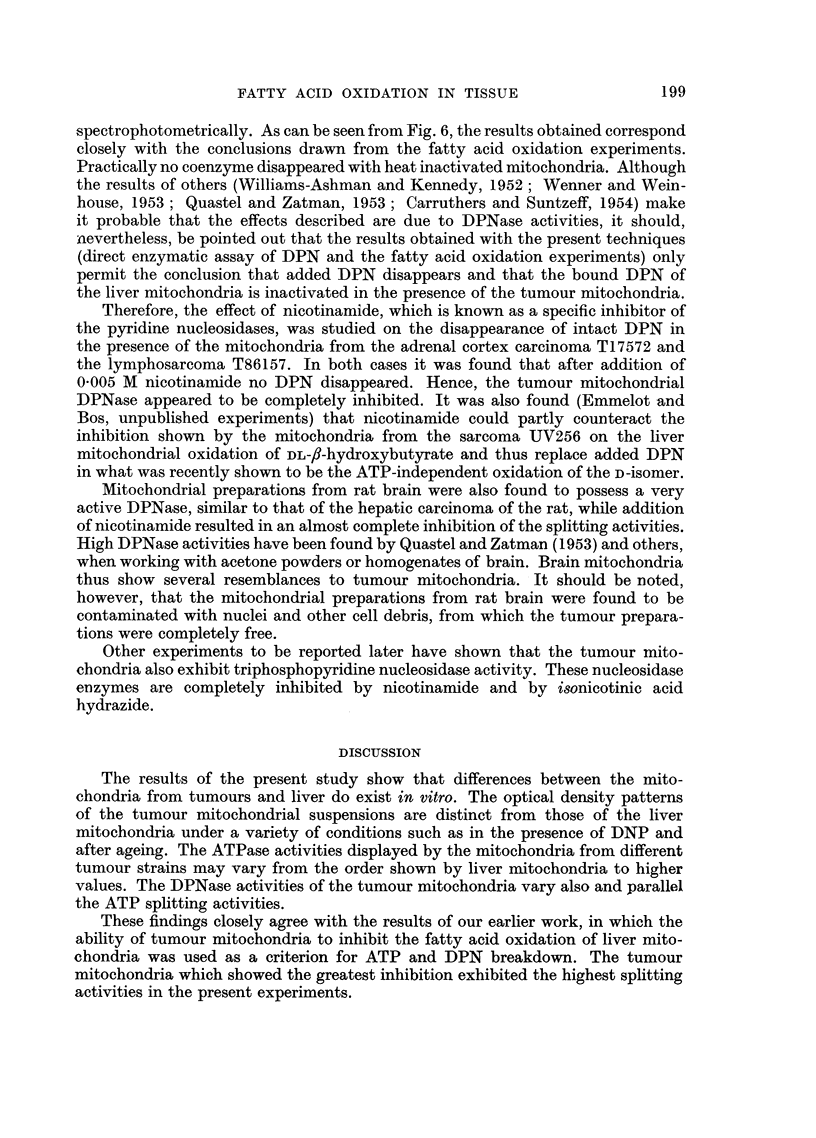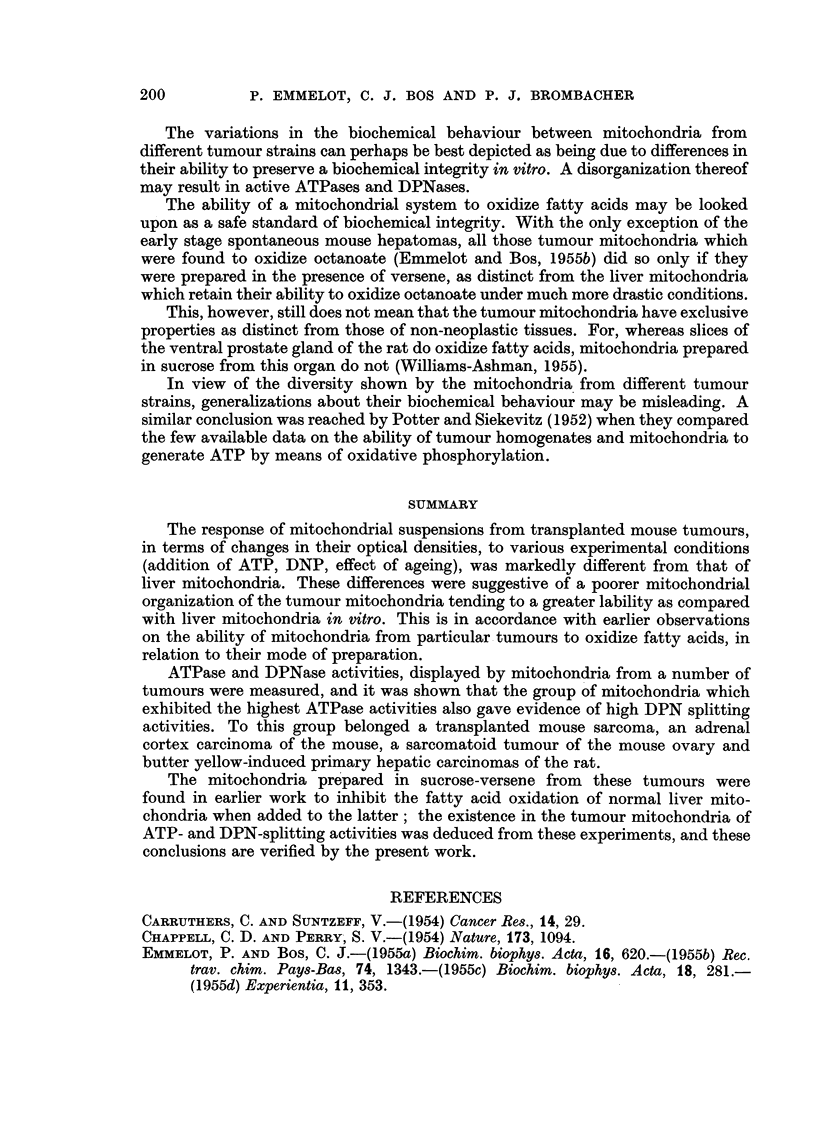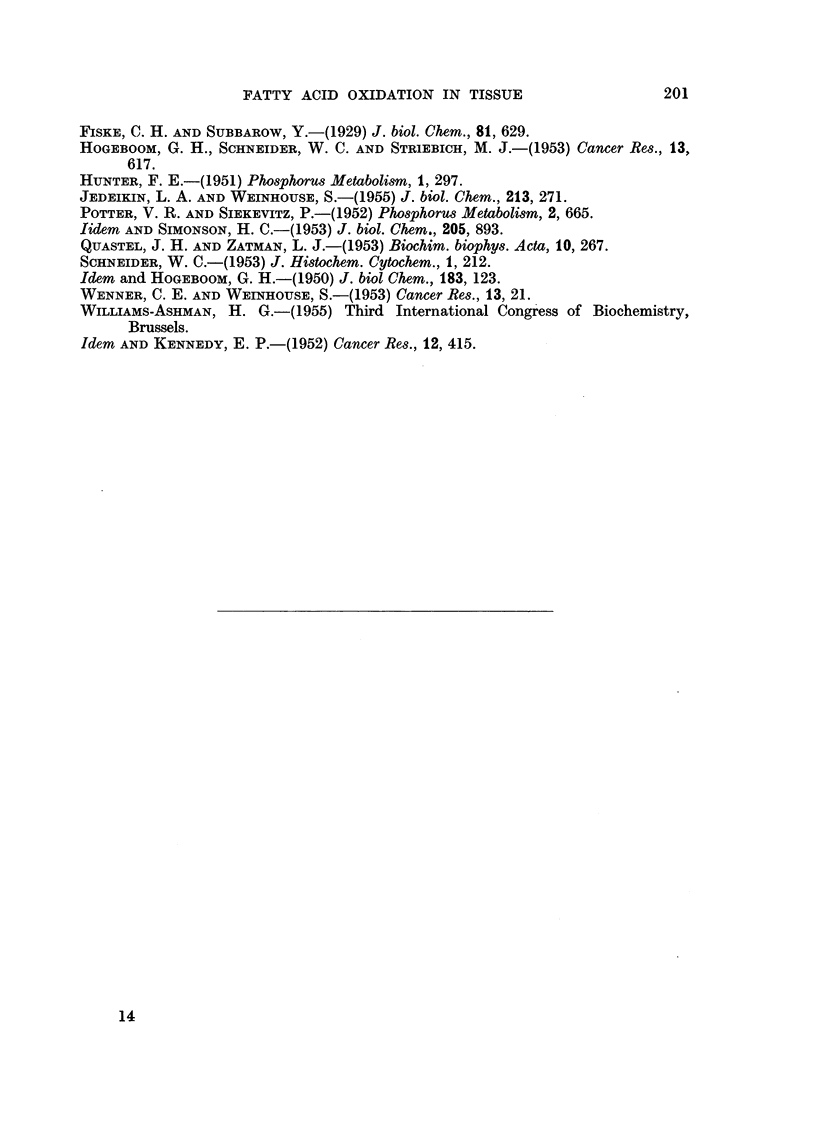# Fatty Acid Oxidation in Normal and Neoplastic Tissues

**DOI:** 10.1038/bjc.1956.22

**Published:** 1956-03

**Authors:** P. Emmelot, C. J. Bos, P. J. Brombacher


### FATTY ACID OXIDATION IN NORMAL AND NEOPLASTIC TISSUES

### ENZYMATIC ACTIVITIES AND OPTICAL DENSITIES OF MITOCHONDRIAL SUSPENSIONS PREPARED FROM NORMAL AND NEOPLASTIC TISSUES OF THE MOUSE.

P. EMMELOT, C. J. BOS AND P. J. BROMBACHER

*From the Department of Biochemistry, Antoni van Leeuwenhoek-Huis, The Netherlands Cancer Institute, Amsterdam, the Netherlands*

Received for publication December 19, 1955

IN earlier publications (Emmelot and Bos, 1955*a*, 1955*b*, 1955*c*) experiments were described which indicated that the mitochondria from different tumour strains of the mouse were markedly at variance with each other in the extent to which their adenosine triphosphate and diphosphopyridine nucleotide splitting enzymes (ATPases and DPNases) were active *in vitro*. That these splitting activities might be inherent to tumour mitochondria was deduced from a study on the inhibition of octanoate and DL-$\beta$-hydroxybutyrate oxidation of normal liver mitochondria in the presence of tumour mitochondria. In the present communication more direct evidence on the differences in the tumour mitochondrial ATPase and DPNase activities is presented. At the same time, results of other experiments are described in which a related aspect of mitochondrial behaviour was studied, i.e. the changes in optical density of mitochondrial suspensions in the presence and the absence of ATP and 2 : 4-dinitro phenol (DNP). These experiments were performed in the hope that they would furnish additional information on the properties of the tumour mitochondria in comparison with those of normal liver, since the responsiveness of mitochondria to the addition of ATP and DNP might be governed by the extent of biochemical integrity exhibited by the mitochondria *in vitro*. Biochemical integrity is best illustrated by the intact oxidative and phosphorylative systems of freshly prepared mitochondria from liver with their high P/O quotients, latent ATPase and DPNase activities and, consequently, their ability to oxidize fatty acids (Schneider, 1953; Hogeboom, Schneider and Striebich, 1953).

### EXPERIMENTAL

The tumours studied were well established specimens transplanted in our Institute; particulars of tumours and mice were given in the preceding communication of this series (Emmelot and Bos, 1955*b*). The preparation of the mitochondria for the optical density tests and the medium (*tris* buffer, KCl, versene, $Mg^{2+}$, ATP) used have been adapted from Chappell and Perry (1954). Incubations were carried out in 5 ml. of the same medium without versene and ATP. The amount of mitochondria present varied from 0·7–0·9 mg. nitrogen determined with a micro-Kjeldahl procedure. After 15 minutes at room-temperature, 0·1 ml. 0·1 M ATP was added; DNP, when present, was in a final concentration of $10^{-4}$. M.

Optical densities were read at 520 m$\mu$, with a Unicam spectrophotometer. At 25 and 45 minutes after the introduction of ATP 0·5 ml. of the suspension was deproteinized and used for the determination of inorganic phosphate ($P_i$) (Fiske and Subbarow, 1929). Data were calculated as $\mu$-moles $P_i$ per 1 mg. mitochondrial nitrogen.

Aged mitochondria were obtained by incubation of fresh mitochondria, isolated in the complete medium, in the absence of ATP, $Mg^{2+}$ and versene for one hour at 37° C. After this period, 0·2 ml. portions of this suspension were pipetted into 4·8 ml. of *tris*-KCl medium containing $Mg^{2+}$. Optical densities were then read in the presence and absence of ATP and or DNP. Mitochondria were present in amount corresponding to the afore-mentioned nitrogen content. In other experiments $Mg^{2+}$ 0·005 M was also added during the pre-incubation at 37° C.

*Measurements of ATPase activity* (as given in Fig. 3 and Tables III, IV and V) were made as follows. A mitochondrial suspension containing 2–3 mg. N per ml. was freshly prepared as described and 0·1 ml. was added to 1·7 ml. of a *tris*-KCl-medium containing $Mg^{2+}$ (without versene and ATP) ; 0·1 ml. 0·2 M ATP was also added. Incubation was at 27° C. with shaking, in air. The reaction was stopped by addition of 0·1 ml. 50 per cent trichloroacetic acid. After centrifugation $P_i$ was determined in an aliquot of the clear supernatant.

*Measurements of DPNase activity.*—Mitochondria were prepared in 0·25 M sucrose containing 0·001 M versene according to Schneider and Hogeboom (1950) (the nuclear fraction was not resuspended) and suspended in 0·013 M phosphate buffer (pH 7·4). The resulting suspension contained from 2 to 3 mg. mitochondrial N and 0·3 ml. of this suspension was pipetted into the reaction flasks, which contained 0·3 ml. of a 0·07 M phosphate buffer (pH 7·4), 0·1 ml. 0·16 M 95 per cent DPN (obtained from C. F. Boehringer u.S, Mannheim), and 0·9 ml. water.

Incubation was at 27° C. with shaking, in air. The reaction was stopped by rapid cooling and the mitochondria were centrifuged down at 15,000 $\times$ g for 10 minutes at 0° C. A 0·5 ml. aliquot of the clear supernatant was then pipetted into the cell of the spectrophotometer (Unicam SP500) which contained 0·1 ml. 10 per cent ethanol, 1·5 ml. phosphate buffer (0·07 M pH 7·4) and 0·8 ml. $H_2O$. The optical density at 340 m$\mu$. was adjusted to 100 per cent transmission. After addition of 0·1 ml. of a dilute solution of crystalline alcohol dehydrogenase (also obtained from C. F. Boehringer u.S, Mannheim) (the 30 mg./ml. suspension was diluted 1 : 500 with water), the extinction of the resulting DPNH was read at 340 m$\mu$. and corrected for the addition of the enzyme (Jedeikin and Weinhouse, 1955). The amount of DPNH is calculated with the aid of a curve constructed with the optical densities of known amounts of DPNH formed from DPN by the same procedure.

### RESULTS

### *Experiments with liver mitochondria*

The effect of ATP addition on the optical densities of two different liver mitochondrial suspensions, prepared in the KCl-*tris*-versene medium containing $Mg^{2+}$ and incubated with and without DNP in the absence of versene, are plotted in Fig. 1 ; *a, b, c* and *d*.

In the presence of DNP the fall in optical density of the suspensions to which no ATP was added (b, d ●—— ●) was found to be much steeper, and the maximum of the curve when ATP was added (b, d ×——×), lower than with the suspensions incubated in the absence of DNP (a, c ●——● and ×——×, respectively). The dotted line in Fig. 1*b* shows the course of the optical densities occasionally encountered after ATP addition in experiments, which otherwise gave similar results.

FIG. 1.—Optical densities of liver mitochondria, in the presence and absence of ATP and/or 2 : 4-dinitrophenol (DNP) : and $\mu$-moles $P_i$ split from ATP per 1 mg. mitochondrial N. Abscissa, time in minutes. ●——● controls without ATP ; left part of figure in the absence of DNP, right part in the presence of DNP. ×——× with ATP added ( ↓ ) ; *ditto*. Mg. N present per tube : 0·81 first experiment, 0·67 second experiment.

TABLE I.—*ATPase Activity of Mitochondria from Mouse Livers (C57 Black) in the Presence and Absence of* 2 : 4-*dinitrophenol (DNP)*

Isolation in *tris*-KCl-versene, incubation in *tris*-KCl medium.
Total number of experiments 8.

| DNP. | $\mu$-Moles $P_i$/mg. N released after minutes. 25 | 45 |
|---|---|---|
| — . | 7·1<br>(5·0–10·0) | 10·9<br>(6·0–13·0) |
| + . | 11·0<br>(5·8–13·4) | 13·0<br>(6·8–15·2) |

A comparison of the amounts of inorganic phosphate ($\mu$-moles $P_i$ per 1 mg. mitochondrial nitrogen) liberated to the medium 25 and 45 minutes after addition of ATP (cf. Table I), illustrates the well known effect of DNP in raising the " high energy " phosphate cleavage by mitochondria (Hunter, 1951 ; Potter, Siekevitz and Simonson, 1953). Judging from the results of our series of experiments, of which two examples were illustrated in Fig. 1, it may appear that ATP addition evoked the greatest response with liver mitochondria possessing the most active ATPases.

When, however, the liver mitochondria were aged before use, by pre-incubation in the KCl-*tris* medium in the absence of ATP, $Mg^{2+}$ and versene, ATP addition consistently caused a very significant rise in the optical density of these suspensions (Fig. 2*a*). This rise in the optical density of the aged mitochondrial suspensions from liver closely resembles that of the fresh mitochondria from pigeon breast

FIG. 2.—Optical densities of liver mitochondria after pre-incubation (*a* in the absence, *b* in the presence of 0·005 M $Mg^{2+}$) : Effect of ATP addition ( ↓ ) in the absence of DNP ( × —— × ), and in the presence of DNP ( × - - - × ), as compared with the appropriate controls (a : curve ● - - - ● identical with ● —— ● ; b with DNP same curves as without). The optical densities of the fresh mitochondrial suspension, containing the same amount of N (0·67 mg.), was 0·480 (520 mμ.) at 0 min.

muscle studied by Chappell and Perry (1954). The effect of the pre-incubation on the activity of the mitochondrial ATPase as shown by the μ-moles $P_i$ appearing in the medium, was 6·6 for fresh and 8·2 for aged mitochondria. In another experiment 5·8 and 9·3 μ-moles $P_i$ were found respectively, but in a third no activation was found (10 and 10·3).

When the liver mitochondria were freshly prepared and examined by phase contrast microscopy practically no aggregation of mitochondrial bodies could be seen, but after 60 minutes at room temperature some aggregation had taken place. The optical densities of the pre-incubated mitochondria were always much lower than those of freshly prepared suspensions containing the same amount of mitochondrial nitrogen. This may be due not only to a swelling of the mitochondria but very probably also to an aggregation of the particles. Upon ATP addition there was some indication that the extent of this mitochondrial aggregation diminished, while at the same time the individual mitochondria regained a more compact appearance.

The presence of DNP in the suspending medium of the aged liver mitochondria did not result in the steeper fall of optical densities such as was shown by their

FIG. 3.—ATPase activities of liver mitochondria as a function of mitochondrial concentration (μ moles $P_i$ split from ATP calculated per mg. N).

●——● 0·38⁶ mg. N/tube. ×——× 0·12⁹ mg. N/tube.

○——○ 0·19³ „ „ ▲——▲ 0·09⁷ „ „

The mitochondria used in this experiment were obtained from livers of Odz × dz mice. It was found that these particles sometimes showed much higher ATPase activities than those of the other mouse strains (compare Table IV) and were unable to oxidize fatty acids under the standard conditions. This figure illustrates such an example. (For conditions see *Experimental.*)

fresh counterparts. Neither was the ATPase found to be activated by DNP in two experiments (8·2 and 8·7 ; 9·3 and 9·3 $\mu$-moles $P_i$ released with and without DNP present), but an activation was found in the third experiment from 10·3 to 12·3 $\mu$-moles $P_i$. The response of the aged mitochondria to ATP addition in the presence of DNP was, however, markedly slower and less than in the absence of DNP.

When the liver mitochondria were pre-incubated *in the presence of* $Mg^{2+}$—an activator of the mitochondrial ATPase—the response in terms of optical densities to ATP was almost, and that to DNP was completely absent (Fig. 2*b*) ; the ATPase was found to be activated further (from 8·2 to 10·8 and 9·3 to 11·3 $\mu$-moles $P_i$, respectively, as compared after ageing in the absence of $Mg^{2+}$).

In the experiments with liver mitochondria and especially with the tumour mitochondria it was not always possible to use the same amount of mitochondria. Therefore the ATPase activity was measured as a function of mitochondrial concentration, using liver mitochondria freshly prepared in the complete KCl-*tris*-versene medium and diluted to 1/2, 1/3 and 1/4 of the original volume. The results, expressed as $\mu$-moles $P_i$ released per 1 mg. mitochondrial nitrogen (Fig. 3), show that the smaller the concentration of the mitochondria, the greater the amount of $P_i$ appearing in the medium, This was also found in other instances, for example with a combination of liver and tumour mitochondria. (The data in the tables are not corrected for this " concentration-effect ".) Although the exact reason for this phenomenon is not known to us, it may perhaps be connected with mitochondrial aggregation.

*Experiments with tumour mitochondria*

In order to study the variations in optical density of tumour mitochondrial suspensions in the presence and absence of ATP and DNP, together with their ATPase activities, four transplanted tumours of the mouse were selected : T26554 an interstitial cell carcinoma of the testis ; T5441, a granulosa cell tumour of the ovary ; UV256, a sarcoma ; and T17572, a carcinoma of the adrenal cortex.

The mitochondria of these tumours differ in their biochemical behaviour as judged from the inhibitory effect which they produce on the fatty acid oxidation of normal liver mitochondria. The sucrose-versene mitochondria of the first two tumours do not interfere with the fatty acid oxidation of liver mitochondria ; the tumour mitochondria *per se* are even capable of oxidizing octanoate (low ATPase and DPNase activities). However, under other conditions of preparation, the mitochondria from T5441 are found to show some differences from those of T26554. But the mitochondria from the sarcoma and the adrenal cortex carcinoma, when prepared in sucrose-versene, completely inhibit the fatty acid oxidation of the liver mitochondria (high ATPase and DPNase activities ; cf. Emmelot and Bos, 1955*b*). The results of Fig. 4 show that the same order in ATPase activities is found under the present conditions by comparing the amounts of $P_i$ released from ATP into the suspending medium with the conclusions drawn from the " inhibition assay ".

Furthermore it is seen (Fig. 4) that the tumour mitochondria do not respond to DNP in the same way as the liver mitochondria. First, DNP did *not* enhance the ATPase activities of the mitochondria from the transplanted tumours to any significant extent, not even with those mitochondria (e.g. from T26554) which exhibit an ATPase activity of the same order as that found for the liver mitochondria. Secondly, the optical density-time curve of the tumour mitochondria does *not* show a steeper fall in the presence of DNP than in its absence. Hence it would appear as if DNP had more or less lost its point of attack on the tumour mitochondria. Nevertheless, DNP abolishes the retarding effect shown by ATP on the fall in optical density of the tumour mitochondrial suspensions in a few

FIG. 4.—Optical densities of tumour mitochondria, in the absence and presence of ATP and, or DNP, and $\mu$ moles $P_i$ split from ATP per 1 mg. mitochondrial N. ●——● and ×——× compare Fig. 1. : : visible agglutination. Mg. N present per tube : T26554 0·87 and 0·78, T5441 0·85, UV256 0·79, and T17572 0·87.

cases (compare T26554). In view of the known action of DNP to evoke the latent ATPase activity of fresh liver mitochondria, and the absence of a DNP-effect on the ATPase of mitochondria which are already activated, the findings reported may indicate that under the particular conditions of the present experiments, the fresh tumour mitochondria are already in such a state as prevails when fresh liver mitochondria are " damaged " as, for example, by ageing in the presence of $Mg^{2+}$. It was therefore not unexpected to find that ageing of the tumour mitochondrial suspensions did not change their optical density after addition of ATP or DNP, nor did it activate their ATPase.

It has been found that sucrose isolated mitochondria from liver when aged in KCl-phosphate buffer at 37° C.—a condition under which resting mitochondria pass soon into a " damaged " stage—are capable of inhibiting the fatty acid oxidation of their fresh counterparts (Emmelot and Bos, 1955*b*). The behaviour of such treated liver mitochondria resembles that of the fresh sucrose mitochondria of most tumours studied by us. These, together with the other findings, had led us to the opinion that the factor(s) responsible for the inhibition of fatty acid oxidation in the combined system of liver and tumour mitochondria may arise in consequence of an *in vitro* " disorganization " of the integrated biochemical structure of the tumour mitochondria, causing among other effects the activation of latent ATPase and DPNase activities.

In many, but not in all, experiments with the mitochondria from the transplanted tumours, a visible agglutination (indicated on Fig. 4 by : : ) was evident in the absence of ATP. It became apparent between 70–120 minutes' incubation at room-temperature and occurred irrespective whether DNP was present or not. As can be seen from Fig. 4, ATP did always prevent the visible agglutination. As mentioned earlier, phase contrast microscopical observation showed that an aggregation of the mitochondria from liver and tumour occurred sooner or later during incubation. This phenomenon may interfere with an interpretation of the optical density curves in terms of mitochondrial swelling (fall) or shrinkage (rise). A few examinations by phase contrast microscopy showed no marked alterations in the size of the tumour mitochondria in the presence of ATP, whereas such an effect was sometimes noted with the liver mitochondria (for example, Fig. 1*c*).

Mitochondria prepared from "early stage " and " fully developed " spontaneous hepatomas, found in a high incidence in old female (C57 Black × $C_3H_e$) hybrids, were also studied. By using the criteria of the inhibition of fatty acid oxidation, it was argued that during growth of these spontaneous hepatomas certain biochemical changes took place which were found to manifest themselves *in vitro* and could be explained by an increased activity of the mitochondrial ATPase of fully developed as compared with early stage tumours (Emmelot and Bos, 1955*d*). The mitochondria from the latter tumours were from the biochemical point of view much like normal liver mitochondria, those of the fully developed hepatomas resembled closely the mitochondria of T5441. Table II illustrates the phosphate release from ATP by the action of these mitochondria. The ATPase activity observed is in accord with the earlier results. The mitochondria from the spontaneous hepatomas behave similarly to those of the liver in the way their ATPase is activated by DNP. However, the optical density-time relation shown by the

TABLE II.—*ATPase Activity of Mitochondria from Spontaneous Hepatomas* (*C*57 *Black* × $C_3H_e$) $F_1$ ; *Effects of* 2 : 4-*dinitrophenol* (*DNP*)

| Mitochondria from. | mg./N tube. | μMoles $P_i$/mg. N released after minutes. 25 – DNP. | 25 + DNP. | 45 – DNP. | 45 + DNP. |
|---|---|---|---|---|---|
| Spontaneous hepatomas : | | | | | |
| " Early stage " | 0·67 | 9·9 | 13·5 | 13·3 | 14·7 |
| " Fully developed " | 0·75 | 14·2 | 17·4 | 15·8 | 22·0 |

hepatoma mitochondria, incubated in the presence of DNP, was not like that of the liver mitochondria shown in Fig. 1*b* and *d*, but followed that of the other transplanted tumours presented in Fig. 4. Rat brain mitochondria, which had been found (Emmelot and Bos, 1955*b*) to inhibit the octanoate oxidation of the liver mitochondria when added to the latter, had also optical density-time relations similar to those of the transplated tumours. The ATPase activities of brain mitochondria were also much higher than those of the liver mitochondria, as can be seen from Fig. 5.

FIG. 5.—Optical densities of brain mitochondria, in the presence and absence of ATP and, or DNP, and μ moles $P_i$ split from ATP per 1 mg. mitochondrial N (mg. N present per tube : 0·67). ●——● and ×——× ; as in Fig. 1.

TABLE III.—*ATPase Activity of Tumour Mitochondria in the Absence and in the Presence of* 2 : 4-*dinitrophenol* (*DNP*)

Isolation in KCl-*tris*-versene medium, suspension in KCl-*tris* medium. Incubation at 27° C.

| Mitochondria from tumour. | mg. N/ tube. | DNP. | μ-Moles $P_i$/mg. N released after minutes. 5 | 10 | 20 | 30 | 40 |
|---|---|---|---|---|---|---|---|
| T26554 | 0·21 | — | 4·8 | 10·3 | 13·1 | 18·0 | — |
| T5358* | 0·23 | — | 7·5 | 9·0 | 12·0 | 17·1 | 18·1 |
| | 0·17 | — | 8·0 | 10·8 | 12·5 | 15·8 | 16·6 |
| | ,, | + | 6·6 | 8·0 | 11·2 | 13·1 | 16·6 |
| T49985† | 0·32 | — | 6·6 | 9·0 | 13·5 | 19·0 | — |
| | ,, | + | 6·0 | 8·6 | 13·3 | 18·9 | — |
| T28012‡ | 0·19 | — | 7·4 | 11·3 | 16·0 | 20·6 | — |
| | ,, | + | 8·0 | 12·7 | 17·5 | — | — |
| Hepatic carcinoma (rat) ‖ | 0·27 | — | 7·7 | 13·0 | 20·0 | 25·0 | 28·2 |
| T26473‡ | 0·27 | — | 7·0 | 10·3 | 18·4 | 27·0 | — |
| T24202§ | 0·22 | — | 7·6 | 12·4 | 20·6 | 26·6 | — |
| UV256 | 0·25 | — | 9·7 | 13·0 | 19·8 | 26·2 | 29·4 |
| | 0·27 | — | 10·3 | 15·3 | 21·5 | 29·3 | 31·3 |
| T17572 | 0·33 | — | 10·1 | 15·8 | 27·4 | 31·0 | 38·0 |

* Testis tumour. † Mammary carcinoma. ‡ Hepatoma. § Sarcomatoid ovarian tumour.
‖ Primary tumour, induced by butter yellow feeding ; compare : normal part of this rat liver (0·27 mg. N/tube) 5·6, 7·5, 9·3, 10·3 and 11·9 μ moles $P_i$ split from added ATP per 1 mg. mitochondrial N over the same time periods.

The results of further measurements of the ATPase activities of tumour mitochondria, under conditions which were different from the preceding experiments, are given in Table III. Here, again, the mitochondria from the tumours belonging to the same group as the sarcoma and the adrenal cortex carcinoma show the highest activities. Also falling in this class are the mitochondria from the hepatic carcinomas induced by butter yellow feeding in rats (pure strain R Amsterdam), which exhibited an ATPase activity over twice as high as that of the non-neoplastic part of the liver. The results obtained with mitochondria from the livers of three strains of mice (CBA, C57 Black and $C_3H$) were of the same order as those from rat liver (Table IV).

TABLE IV.—*ATPase Activity of Mitochondria from Livers of Three Strains of Mice*

Isolation in KCl-*tris*-versene medium. Incubation KCl-*tris* medium at 27° C.

| Mouse strain. | mg. N/tube. | $\mu$-Moles $P_i$/mg. N released after minutes. 5 | 10 | 20 | 30 |
|---|---|---|---|---|---|
| CBA | 0·30 | 3·3 | 6·8 | 9·3 | 11·7 |
| C57 Black | 0·24 | 3·6 | 7·6 | 9·7 | 13·3 |
| $C_3H$ | 0·33 | 5·4 | 6·8 | 8·7 | 12·3 |
| | 0·28 | 6·6 | 8·4 | 12·3 | 14·3 |

The effect of $Mg^{2+}$ and $Ca^{2+}$ on the ATPase of the mitochondria from the sarcoma UV256 (Table V) clearly shows the activating effect of $Mg^{2+}$ on the tumour mitochondrial ATPase. The activating effect of $Ca^{2+}$ appears to be smaller than that of $Mg^{2+}$.

TABLE V.—*Effect of $Mg^{2+}$ and $Ca^{2+}$ on the ATPase of the Mitochondria from Sarcoma UV* 256

Isolation in the *tris*-KCl medium in the presence of versene (t + v) and in the absence of versene (t — v), and *ditto* of $Mg^{2+}$. Incubation in the absence of versene at 27° C., $Mg^{2+}$ or $Ca^{2+}$ (0·005 M) as indicated.

| Isolation. | $Mg^{2+}$. | $Ca^{2+}$. | mg. N/tube. | $\mu$-Moles $P_i$/mg. N released after minutes. 5 | 10 | 20 | 30 |
|---|---|---|---|---|---|---|---|
| t + v | + | − | 0·29 | 9·1 | 12·6 | 19·5 | 26·5 |
| | − | − | | 6·5 | 7·5 | 10·3 | 13·8 |
| t − v | + | − | 0·26 | 11·8 | 15·3 | 23·8 | 32·3 |
| | − | − | | 6·1 | 7·4 | 10·1 | 12·8 |
| t + v | − | + | 0·26 | 5·5 | 8·1 | 12·1 | 16·4 |
| t − v | − | + | 0·25 | 7·8 | 9·4 | 13·5 | 17·0 |

As was shown earlier, DPNase activaties were also (partly) responsible for the inhibitory effect of tumour mitochondria on the fatty acid oxidation of liver mitochondria. These experiments (Emmelot and Bos, 1955*c*) showed that, with sucrose-versene prepared mitochondria, high activities were inherent to the following tumours : T17572, UV256, T24202 (a sarcomatoid tumour of the mouse ovary), butter yellow induced primary carcinomas of the rat liver and, sometimes, T86157 a lymphosarcoma. No such activities could be ascribed to, among others, T5441, T26554, T5358 (an interstitial carcinoma of the mouse testis) and T28012 (a mouse hepatoma).

These variations between the mitochondria of different tumour strains have now also been studied more directly. Tumour mitochondria were prepared in sucrose-versene and incubated up to thirty minutes in a phosphate buffer in the presence of DPN. At fixed time intervals the DPN left was introduced into the system alcohol-alcohol dehydrogenase and the resulting DPNH was measured

FIG. 6.—DPNase activities of tumour mitochondria (amount of DPN disappearing calculated as per cent of DPN added).

△——△ Primary hepatic carcinoma (rat).

■——■ T17572.

□——□ UV256.

○——○ T28012.

×——× T26554.

●——● T5441.

▲——▲ T5358.

+——+ T24202.

Descriptions of the tumours used are given in the text.

spectrophotometrically. As can be seen from Fig. 6, the results obtained correspond closely with the conclusions drawn from the fatty acid oxidation experiments. Practically no coenzyme disappeared with heat inactivated mitochondria. Although the results of others (Williams-Ashman and Kennedy, 1952 ; Wenner and Weinhouse, 1953 ; Quastel and Zatman, 1953 ; Carruthers and Suntzeff, 1954) make it probable that the effects described are due to DPNase activities, it should, nevertheless, be pointed out that the results obtained with the present techniques (direct enzymatic assay of DPN and the fatty acid oxidation experiments) only permit the conclusion that added DPN disappears and that the bound DPN of the liver mitochondria is inactivated in the presence of the tumour mitochondria.

Therefore, the effect of nicotinamide, which is known as a specific inhibitor of the pyridine nucleosidases, was studied on the disappearance of intact DPN in the presence of the mitochondria from the adrenal cortex carcinoma T17572 and the lymphosarcoma T86157. In both cases it was found that after addition of 0·005 M nicotinamide no DPN disappeared. Hence, the tumour mitochondrial DPNase appeared to be completely inhibited. It was also found (Emmelot and Bos, unpublished experiments) that nicotinamide could partly counteract the inhibition shown by the mitochondria from the sarcoma UV256 on the liver mitochondrial oxidation of DL-$\beta$-hydroxybutyrate and thus replace added DPN in what was recently shown to be the ATP-independent oxidation of the D-isomer.

Mitochondrial preparations from rat brain were also found to possess a very active DPNase, similar to that of the hepatic carcinoma of the rat, while addition of nicotinamide resulted in an almost complete inhibition of the splitting activities. High DPNase activities have been found by Quastel and Zatman (1953) and others, when working with acetone powders or homogenates of brain. Brain mitochondria thus show several resemblances to tumour mitochondria. It should be noted, however, that the mitochondrial preparations from rat brain were found to be contaminated with nuclei and other cell debris, from which the tumour preparations were completely free.

Other experiments to be reported later have shown that the tumour mitochondria also exhibit triphosphopyridine nucleosidase activity. These nucleosidase enzymes are completely inhibited by nicotinamide and by *iso*nicotinic acid hydrazide.

### DISCUSSION

The results of the present study show that differences between the mitochondria from tumours and liver do exist *in vitro*. The optical density patterns of the tumour mitochondrial suspensions are distinct from those of the liver mitochondria under a variety of conditions such as in the presence of DNP and after ageing. The ATPase activities displayed by the mitochondria from different tumour strains may vary from the order shown by liver mitochondria to higher values. The DPNase activities of the tumour mitochondria vary also and parallel the ATP splitting activities.

These findings closely agree with the results of our earlier work, in which the ability of tumour mitochondria to inhibit the fatty acid oxidation of liver mitochondria was used as a criterion for ATP and DPN breakdown. The tumour mitochondria which showed the greatest inhibition exhibited the highest splitting activities in the present experiments.

The variations in the biochemical behaviour between mitochondria from different tumour strains can perhaps be best depicted as being due to differences in their ability to preserve a biochemical integrity *in vitro*. A disorganization thereof may result in active ATPases and DPNases.

The ability of a mitochondrial system to oxidize fatty acids may be looked upon as a safe standard of biochemical integrity. With the only exception of the early stage spontaneous mouse hepatomas, all those tumour mitochondria which were found to oxidize octanoate (Emmelot and Bos, 1955*b*) did so only if they were prepared in the presence of versene, as distinct from the liver mitochondria which retain their ability to oxidize octanoate under much more drastic conditions.

This, however, still does not mean that the tumour mitochondria have exclusive properties as distinct from those of non-neoplastic tissues. For, whereas slices of the ventral prostate gland of the rat do oxidize fatty acids, mitochondria prepared in sucrose from this organ do not (Williams-Ashman, 1955).

In view of the diversity shown by the mitochondria from different tumour strains, generalizations about their biochemical behaviour may be misleading. A similar conclusion was reached by Potter and Siekevitz (1952) when they compared the few available data on the ability of tumour homogenates and mitochondria to generate ATP by means of oxidative phosphorylation.

### SUMMARY

The response of mitochondrial suspensions from transplanted mouse tumours, in terms of changes in their optical densities, to various experimental conditions (addition of ATP, DNP, effect of ageing), was markedly different from that of liver mitochondria. These differences were suggestive of a poorer mitochondrial organization of the tumour mitochondria tending to a greater lability as compared with liver mitochondria *in vitro*. This is in accordance with earlier observations on the ability of mitochondria from particular tumours to oxidize fatty acids, in relation to their mode of preparation.

ATPase and DPNase activities, displayed by mitochondria from a number of tumours were measured, and it was shown that the group of mitochondria which exhibited the highest ATPase activities also gave evidence of high DPN splitting activities. To this group belonged a transplanted mouse sarcoma, an adrenal cortex carcinoma of the mouse, a sarcomatoid tumour of the mouse ovary and butter yellow-induced primary hepatic carcinomas of the rat.

The mitochondria prepared in sucrose-versene from these tumours were found in earlier work to inhibit the fatty acid oxidation of normal liver mitochondria when added to the latter ; the existence in the tumour mitochondria of ATP- and DPN-splitting activities was deduced from these experiments, and these conclusions are verified by the present work.

### REFERENCES

CARRUTHERS, C. AND SUNTZEFF, V.—(1954) *Cancer Res.*, **14**, 29.
CHAPPELL, C. D. AND PERRY, S. V.—(1954) *Nature*, **173**, 1094.
EMMELOT, P. AND BOS, C. J.—(1955*a*) *Biochim. biophys. Acta*, **16**, 620.—(1955*b*) *Rec. trav. chim. Pays-Bas*, **74**, 1343.—(1955*c*) *Biochim. biophys. Acta*, **18**, 281.—(1955*d*) *Experientia*, **11**, 353.

Fiske, C. H. and Subbarow, Y.—(1929) *J. biol. Chem.*, **81**, 629.
Hogeboom, G. H., Schneider, W. C. and Striebich, M. J.—(1953) *Cancer Res.*, **13**, 617.
Hunter, F. E.—(1951) *Phosphorus Metabolism*, **1**, 297.
Jedeikin, L. A. and Weinhouse, S.—(1955) *J. biol. Chem.*, **213**, 271.
Potter, V. R. and Siekevitz, P.—(1952) *Phosphorus Metabolism*, **2**, 665.
*Iidem* and Simonson, H. C.—(1953) *J. biol. Chem.*, **205**, 893.
Quastel, J. H. and Zatman, L. J.—(1953) *Biochim. biophys. Acta*, **10**, 267.
Schneider, W. C.—(1953) *J. Histochem. Cytochem.*, **1**, 212.
*Idem* and Hogeboom, G. H.—(1950) *J. biol Chem.*, **183**, 123.
Wenner, C. E. and Weinhouse, S.—(1953) *Cancer Res.*, **13**, 21.
Williams-Ashman, H. G.—(1955) Third International Congress of Biochemistry, Brussels.
*Idem* and Kennedy, E. P.—(1952) *Cancer Res.*, **12**, 415.